# Supplementary material for: Genomic diversity of diarrheagenic multidrug-resistant Escherichia coli across asymptomatic children and livestock in Nairobi, Kenya
Source: PLOS Glob Public Health. 2026 Apr 1;6(4):e0005644. doi: 10.1371/journal.pgph.0005644 (PMC13042725; doi:10.1371/journal.pgph.0005644)
Supplement: S1 Text — (DOCX) [file pgph.0005644.s003.docx]

## **S1 File**

## **Inclusion and exclusion criteria**

We obtained a complete list of all active community health volunteers (CHVs) within two wards (Uthiru/Ruthimitu and Riruta) from the Dagoretti South Sub-County health offices. Each CHV represented ~100 households (HH). We used random number generator to select 100 CHVs from the list. Out of the selected 100, 90 CHVs were available to participate in the study. Each CHV provided a complete list of all HH that had at least one child aged 6-24 months within their cluster at the time (sampling one presumptive healthy child per HH). We randomly selected 5-7 HH from each of the 90 CHVs totaling to 585 HH. We included HH where adult caregivers gave informed consent to participate in the study.

## **Sample size calculation**

The total count of stool samples from children included in this study was determined using the formula by Dohoo, 2003 [1], n= {(z α/2)^2^ x P (1-P)}/(α/2)^2^. The sample size of 500 households was calculated to enable the detection of the true prevalence of a pathogen in children at any value (0-100%), with a precision of 5%, allowing an attrition rate of 20%. This was calculated using an assumed detection prevalence of 50%. This large sample size is important as previous studies have shown that prevalence of individual enteric pathogens may vary widely - the recently published SHINE trial reports rates of *Shigella* at 1.7% and rates of EAEC at 71.3% measured from the stool of 6-month-old infants in rural Zimbabwe [2].

All livestock in the recruited households were sampled purposively. It was anticipated that 15% of households (n=75) would be keeping livestock in the urban area [3]. Using the information from [3] study conducted in Dagoretti and Korogocho, it was estimated that 10% of the households would be keeping poultry, 6% would be keeping cattle, and 60 goats/sheep and 15 pigs would be kept among the 500 HH. A total of 585 households were recruited from the two wards to adjust for the anticipated dropout rate of the study participants.

## **Data collection**

### **Sample collection and transportation**

We recruited seven registered nurse enumerators whom we trained on participant consenting and sample collection. These were paired with the CHVs to help locate the recruited households. With consent, a household survey preloaded on ODK Collect application was administered. The nurse enumerators would notify the veterinary technicians if the households kept livestock. The veterinary technicians would then visit the households and collect fecal samples.

### **Stool sample collection from children**

Nurse enumerator would give four disposable diapers in a labeled sterile zip lock bag to the caregiver. The nurse enumerator demonstrated to the caregivers how the diaper was to be put on the child. They were instructed to leave the diaper on the child until such a time when the child would defecate or when the diaper would be full of urine. Upon producing stool, the diaper was to be folded carefully into a ball without touching the inside, placed inside the zip lock bag, and kept safe awaiting collection by the CHV or nurse enumerator. In cases where no stool was produced, this process would be repeated early the next morning once the child woke up. Collected diapers were placed inside a cooler box, kept at 4^o^C, and transported to ILRI laboratories within 6 hours of collection. In total, 540 stool samples were obtained.

### **Food sample collection**

The nurse enumerator asked the caregiver to give them a sample of food they would feed to the child if they were hungry (ready-to-eat, RTE). The caregiver was asked to fill up a 50ml falcon tube to at least halfway with the food in whichever way they would serve it to the child. The falcon tube was then capped tightly and labelled with ward ID, household ID, date, and type of food. This was then placed inside a zip lock bag, labelled the same way, and placed inside a cooler box. The type of food sampled was recorded on a tablet. The sample was then transported to ILRI laboratory within 6 hours for processing. A total of 585 food samples were collected from the households.

In phase 2 of the study involving food value chains (trace back), we selected a subset of the households (109/585) to participate in household food preparation observations. The 109 households were purposively selected where the HH were divided based on the children age groups (6-12 months, 13-18 months, and 19-24 months) reflecting diversity in household survey responses including socio-economic status, types of foods consumed by children, livestock keeping, et cetera. The caregivers from the 109 selected HH were accompanied to the vendors from whom they purchased the four priority foods selected based on what was most eaten and was at the highest risk of contamination. Once these were purchased, a portion was taken to represent vendor sample (98 vendors samples) and the rest used in food preparation observation after which another sample was taken from the prepared food for processing in the laboratory.

The vendors’ behavior and manner of handling foods was observed and recorded, with their consent. Since seasonal differences and preferences informed the vendors’ choice of suppliers and local producers, this information was used to select which suppliers/local producers to recruit. Fruits, vegetables, milk and “matoke” (mashed bananas) were sampled from local producers, while rice, porridge and ugali flour were sampled from suppliers. These foods were sampled from 67 producers/suppliers and their ways of handling foods observed and recorded in the questionnaires as done for the vendors.

### **Stool sample collection from livestock**

This was incidental to the household sampling of children stools and foods. The veterinary technicians visited the households reported by the enumerators to be keeping livestock (cattle, sheep, goats, pigs, and poultry). With consent, the veterinary technicians collected livestock fecal samples. Before sampling, the animals were allowed to stand quietly for about half an hour after mustering/yarding before sampling. The animal was then restrained in a standing position. Using gloved hand, one finger was inserted into the rectum and where feces were present, these were scooped out by gently hooking with a fingertip and withdrawing the finger. Feces were placed in a 50mL falcon tube labelled with household ID, animal ID and date and then placed into a zip lock bag labelled the same way. Where no feces were palpable in the rectum, the finger was rotated to stimulate the anal reflex. Where no feces were palpable after 20-30 seconds, the animal was left for 30 minutes, and the process repeated with a fresh pair of gloves. The fecal samples were then placed inside a cooler box and transported within 6 hours to ILRI laboratory for processing. For chickens (poultry), rectal swabs of up to 5 chickens per household were taken and pooled as one sample. A total of 296 livestock samples were obtained from 95 households.

### **Pathotyping of *E. coli***

Enterotoxigenic *E. coli* (ETEC) presence was defined by the detection of heat-stable (stl) and/or heat labile (elt) toxins. Enteropathogenic *E. coli* (EPEC) was defined by the detection of bfp and eae genes (typical EPEC) and presence of eae and absence of bfp (atypical EPEC). Enteroaggregative *E. coli* (EAEC) presence was defined by detection of aafII and/or pic, while Enteroinvasive *E. coli* (EIEC) was defined by detection of ipaH and virF genes. Diffusely adhesive *E. coli* (DAEC) was defined by the detection of daaE gene. Any *E. coli* strain that produced stx1 and/or stx2 was termed Shiga toxin-producing *E. coli* (STEC) with or without eae gene.

## **References**

1. Ian Dohoo, Wayne Martin, Stryhn Henrik. Veterinary Epidemiologic Research2003.

2. McQuade Elizabeth T. Rogawski, Platts-Mills James A., Gratz Jean, Zhang Jixian, Moulton Lawrence H., Mutasa Kuda, et al., Impact of water quality, sanitation, handwashing, and nutritional interventions on enteric infections in rural zimbabwe: The sanitation hygiene infant nutrition efficacy (SHINE) trial. Journal of Infectious Diseases 221 (2020) 1379-1386, <https://doi.org/10.1093/infdis/jiz179>.

3. Dominguez-Salas Paula, Alarcón P., Häsler B., Dohoo I. R., Colverson K., Kimani-Murage E. W., et al., Nutritional characterisation of low-income households of Nairobi: socioeconomic, livestock and gender considerations and predictors of malnutrition from a cross-sectional survey. BMC Nutrition 2 (2016) 1-20, <https://doi.org/10.1186/s40795-016-0086-2>.
