# Supplementary material for: Genomic diversity of diarrheagenic multidrug-resistant Escherichia coli across asymptomatic children and livestock in Nairobi, Kenya
Source: PLOS Glob Public Health. 2026 Apr 1;6(4):e0005644. doi: 10.1371/journal.pgph.0005644 (PMC13042725; doi:10.1371/journal.pgph.0005644)
Supplement: Attachment — Submitted filename: Response to Reviewers.docx [file pgph.0005644.s006.docx]

Response to Editors and Reviewers

Journal Requirements:

1. Please amend your detailed Financial Disclosure statement. This is published with the article. It must therefore be completed in full sentences and contain the exact wording you wish to be published.

i. Please clarify all sources of financial support for your study. List the grants, grant numbers, and organizations that funded your study, including funding received from your institution. Please note that suppliers of material support, including research materials, should be recognized in the Acknowledgements section rather than in the Financial Disclosure. 
ii. State the initials, alongside each funding source, of each author to receive each grant. For example: "This work was supported by the National Institutes of Health (####### to AM; ###### to CJ) and the National Science Foundation (###### to AM)."
iii. State what role the funders took in the study. If the funders had no role in your study, please state: “The funders had no role in study design, data collection and analysis, decision to publish, or preparation of the manuscript.”
iv. If any authors received a salary from any of your funders, please state which authors and which funders.

**Response:** *The financial disclosure has been revised. page 15, line 327-336*

2. Please ensure that your Ethics Statement is available in its entirety at the beginning of your Methods section, under a subheading 'Ethics Statement'.

**Response:** *This has been moved to the suggested position. Page 12, line 53-58*

3. Please upload separate figure files in .tif or .eps format. Also, remove the figures from your manuscript file but keep the legends.

For more information about figure files please see our guidelines:

<https://journals.plos.org/globalpublichealth/s/figures> 
<https://journals.plos.org/globalpublichealth/s/figures#loc-file-requirements>

**Response:** *This has been done according to the guidelines*

4. We note that your Data Availability Statement is currently as follows: Data is contained within the article.
 
Please confirm at this time whether or not your submission contains all raw data required to replicate the results of your study. Authors must share the “minimal data set” for their submission. PLOS defines the minimal data set to consist of the data required to replicate all study findings reported in the article, as well as related metadata and methods (<https://journals.plos.org/plosone/s/data-availability#loc-minimal-data-set-definition>).
 
 For example, authors should submit the following data:
 
 - The values behind the means, standard deviations and other measures reported;
 - The values used to build graphs;
 - The points extracted from images for analysis.
 
 Authors do not need to submit their entire data set if only a portion of the data was used in the reported study.
 
 If your submission does not contain these data, please either upload them as Supporting Information files or deposit them to a stable, public repository and provide us with the relevant URLs, DOIs, or accession numbers. For a list of recommended repositories, please see <https://journals.plos.org/plosone/s/recommended-repositories>.
 
 If there are ethical or legal restrictions on sharing a de-identified data set, please explain them in detail (e.g., data contain potentially sensitive information, data are owned by a third-party organization, etc.) and who has imposed them (e.g., an ethics committee). Please also provide contact information for a data access committee, ethics committee, or other institutional body to which data requests may be sent. If data are owned by a third party, please indicate how others may request data access.

If the reviewer comments include a recommendation to cite specific previously published works, please review and evaluate these publications to determine whether they are relevant and should be cited. There is no requirement to cite these works unless the editor has indicated otherwise.

**Response:**

The Data Availability Statement has been revised to read: *Raw sequencing reads generated in this study have been deposited in the NCBI Sequence Read Archive (SRA) under BioProject accession PRJNA1337292. The dataset has been made publicly available. Individual SRA accession numbers for each isolate are provided in Supplementary material (S1 Table). All other data supporting the findings of this study are included within the article and its supporting information.*


**Reviewers' comments:**

Reviewer #1:

1. Line 55, which food sample was this?

**Response:** *These were all types of foods consumed by children, for example fruits, vegetables, milk, “matoke” (mashed bananas), rice, porridge and ugali. Page 2, line 72-73 of S1 File*

2. Line 62, specify which CLSI guidelines you used for ASTs.

**Response:** we have specified the following in the text: *CLSI breakpoints for Enterobacterales - Table 2A (32^nd^ Edition) Page 4, line 72*

3. Line 71, did the vendors give consent to have their behaviour and manners recorded and observed?

**Response:** *Yes, the vendors gave consent for this. Page 2, line 69-70 in S1 File)*

4. Line 100, what were the thresholds of identity and length for reproducibility?

**Response**: *≥90% identity, ≥60% coverage. Page 6, line 118*

5. Line 129, could you clarify what this statement, only two STs (ST10 and ST48) were found in both child and livestock.

**Response:** *We have revised the text to read: Only two STs (ST10 and ST48) were shared between children and livestock isolates, while all other STs were exclusive to one host group*. *Page 7, line 151-153*

6. Line 146, was there concordance between phenotypic and genotypic resistance patterns?

**Response:** *We agree that assessing concordance between phenotypic and genotypic resistance is valuable in many genomic surveillance studies. However, in our study the isolates were pre-selected based on MDR phenotype, and so do not represent an unbiased or population-representative sample of E. coli. Because of this pre-selection, concordance analysis would be inherently constrained and potentially misleading, as the dataset is enriched for resistant phenotypes and corresponding resistance determinants. High concordance would therefore be expected and would not meaningfully reflect predictive performance of genotypic resistance detection.*

7. Line 156, were there specific ARGs found in each sample type?

**Response:** *While bla_CTX-M-15_ and bla_OXA-1_ were predominantly detected in isolates from children (and in the single food isolate), the other ARGs were shared across sources, with no clear evidence of source-specific/exclusive resistance patterns. Page 8, line 182-183; page 11, line 252-253, 271-275*
8. Line 178, it would be better to represent the genes encoding virulence factors associated with pathogenic E. coli. you also left out the word WITH.

**Response:** *This has been corrected to read: “Genes encoding virulence factors associated with diarrheagenic E. coli”. Page 10, line 213*

Reviewer #2: Title: Genomic diversity of pathogenic multidrug-resistant Escherichia coli across asymptomatic children and livestock in Nairobi, Kenya - PGPH-D-25-03679

Overall assessment
This is a well-executed genomic epidemiology study addressing an important One Health question in a high-risk peri-urban setting. The integration of WGS, resistome, virulome, and network analyses is a clear strength, and the manuscript is generally well written and contextualized. However, several conceptual and methodological issues limit the strength of causal inference, particularly regarding transmission, pathogenicity, and public health implications. Some claims are over-interpreted given the cross-sectional design and sampling framework, and additional clarification, sensitivity analyses, and reframing are required.

Major comments

1. Selection bias and representativeness of sequenced isolates

Only multidrug-resistant diarrhoeagenic E. coli were selected for sequencing. This creates a strong selection bias that limits interpretation of population structure, AMR prevalence, and virulence burden. Statements about “cryptic reservoirs”, and “community-level pathogenic potential” are not generalizable to community E. coli populations, but only to MDR DEC. I recommend the authors to explicitly acknowledge in the Abstract and Discussion that findings apply only to MDR DEC, not to commensal E. coli, and rephrase claims implying population-wide AMR burden.

**Response:**

*We agree that restricting WGS to MDR DEC introduces selection bias and limits generalizability to the broader community E. coli population. We have revised the* ***Abstract*** *and* ***Discussion*** *sections to explicitly state that our genomic findings apply only to MDR DEC isolates. Claims implying population-wide AMR prevalence, cryptic reservoirs, or community-level pathogenic potential have been rephrased to reflect this restricted scope. These revisions ensure that interpretations are appropriately framed within the context of MDR DEC. A final paragraph has been added to highlight the limitation of this study with respect to bias on sequencing only MDR.*

2. Inference of cross-host transmission is overstated

The manuscript frequently implies cross-host transmission based on shared STs, ARG profiles, and phylogenetic interspersion, but no SNP-distance thresholds or transmission analyses are presented. Shared STs or ARG clusters do not imply recent transmission and the authors themselves note genetic heterogeneity within STs, which weakens transmission claims. The authors should tone down on the language from “frequent cross-host transmission” to “overlapping reservoirs” and add a SNP-distance distribution for isolates sharing STs across hosts, or explicitly state that distances exceed plausible transmission thresholds.

**Response:**

*We agree that shared STs, AMR gene profiles, and phylogenetic interspersion alone do not demonstrate recent cross-host transmission. We have therefore revised the manuscript to replace language implying “frequent cross-host transmission” with wording that reflects overlapping human–food–livestock reservoirs.*

*In addition, we agree that SNP-distance distributions among isolates sharing sequence types can be informative for evaluating recent transmission. However, in the present study, our sampling was cross-sectional and uneven across hosts (59 child isolates vs 17 livestock isolates), and isolates were pre-selected based on an MDR phenotype rather than collected to investigate transmission dynamics. As a result, only a limited number of STs were shared across hosts, and the dataset lacks the density and epidemiological linkage required for robust inference of SNP-based transmission thresholds. We were therefore concerned that generating a SNP-distance distribution could lead to overinterpretation.*

3. Network analysis interpretation is biologically speculative

The MDR “backbone” cluster is interesting, but the manuscript implies plasmid-level stability and ecological centrality without direct plasmid reconstruction. Co-occurrence does not confirm physical linkage and the same genes may co-occur due to historical selection rather than active transmission. I recommend the authors to explicitly state that the network reflects statistical co-occurrence, not confirmed genetic linkage.

**Response:**

*Added in results section: The MDR cluster represents statistical co-occurrence of resistance determinants across isolates and does not imply physical genetic linkage, plasmid carriage, or co-localization on the same mobile element. Page 9-10, line 207-209*

*Discussion section revised: This co-occurrence might indicate the involvement of mobile genetic elements, likely conjugative plasmids, capable of co-transferring multiple resistance determinants in a single event, thereby facilitating the rapid acquisition of a multidrug-resistant phenotype and posing substantial public health concern. Alternatively, this may reflect overlapping or cumulative antibiotic exposures at the individual and community level that sustain selection pressures. Page 13, line 279-283*
4. Food isolate interpretation is disproportionate

One food isolate is described as “pan-resistant” and repeatedly highlighted; n=1 does not permit inference and itt risks overstating food-borne transmission. The authors are advised not to use a single isolate to extrapolate food-chain risks and to consider this as an illustrative case and not evidence.

**Response:**

*We thank the reviewer for this important clarification. We agree that a single food isolate does not permit inference regarding food-borne transmission or food-chain risk. Our intention was not to extrapolate risk from this isolate, but to descriptively report an unusual resistance phenotype observed in this study. We have revised the text to explicitly frame this isolate as an illustrative case and not evidence of food-borne transmission, and we have tempered the language throughout to avoid over-interpretation.*

5. Definition and interpretation of “pathogenic” E. coli

The term “pathogenic E. coli” is used broadly and repeatedly, but many isolates carry individual virulence-associated genes rather than coherent pathotype-defining gene sets. Genes like fimH, csgA, and hlyE are common in commensal strains. Additionally, the VirulenceFinder detects gene presence, not expression or pathogenic potential. My recommendation to the authors is to clarify that results indicate “virulence-associated gene carriage”, not confirmed pathogenicity and to consider reporting complete pathotype profiles per isolate, not just aggregate virulence scores.

**Response:**

*We thank the reviewer for this important clarification regarding terminology. We agree that the presence of individual virulence-associated genes detected by VirulenceFinder does not, on its own, confirm pathogenicity, nor does it indicate gene expression or disease-causing potential.*

*In this manuscript, the term “pathogenic E. coli” was used in reference to isolates that had been previously classified into established diarrheagenic E. coli pathotypes using marker-specific PCR assays targeting canonical pathotype-defining genes, as reported in our earlier publication:* [*https://doi.org/10.1371/journal.pgph.0002594*](https://doi.org/10.1371/journal.pgph.0002594)*. We acknowledge that this distinction was not sufficiently clear in the current manuscript.*

*We have revised the text throughout to replace “pathogenic E. coli” with “diarrheagenic E. coli” where interpretation is based solely on canonical pathotype-defining genes. We have also clarified that VirulenceFinder detects gene presence and does not infer expression or pathogenic potential.*

Minor comments:

1. Methods
• Clarify whether one isolate per sample or multiple colonies were sequenced.

**Response:**

*We thank the reviewer for requesting this clarification. In general, one E. coli isolate per sample was selected for WGS. An exception was made for six child samples in which two distinct pathotypes were identified from the same sample. In these cases, both isolates were multidrug-resistant and were therefore included for WGS, resulting in 12 isolates from six samples. Page 4, line 79-83*

• Justify QUAST thresholds (e.g., GC 50–51% is narrow and may exclude valid E. coli).

**Response:** *GC content was used as a descriptive QC metric to confirm species consistency; no assemblies were excluded solely based on GC content. Page 5, line 101-102*
• State explicitly whether recombination was accounted for in phylogenetic inference.

**Response:**

*Recombination was explicitly accounted for in the phylogenetic inference. Putative recombinant regions were identified and masked using Gubbins prior to phylogenetic tree construction. The phylogeny was therefore inferred from the recombination-free alignment. This has now been clarified in the Methods section of the manuscript. Page 5, line 108-109*
2. Statistics
• PERMANOVA results should report R² values, not just p-values.

**Response:** *The R^2^ value has been reported. Page 6, line 122-124*
• Fisher’s exact tests across many ARGs risk low power with n=17 livestock isolates—acknowledge this.

**Response:** *This has been acknowledged. Page 9, line 192-193*

3. Figures
• Fig 2 would benefit from:
o Scale bar in SNPs

**Response:** *This has been included in Fig 2. Page 7*
o Clear bootstrap support threshold

**Response:** *This has been included in Fig 2. Page 7*
• Fig 3: clarify whether percentages are within-source denominators.

**Response:** *Yes, percentages are calculated within each source group. Page 8, line 190*

4. Data availability
• “Data is contained within the article” is not given an accurate SRA deposition; PRJNA1337292 is non-existent on the SRA database. Update the Data Availability Statement to include accession numbers.

**Response:** *We thank the reviewer for noting this. The raw sequencing reads have been deposited in the NCBI SRA under BioProject accession PRJNA1337292. The dataset is currently under controlled release and accessible via the NCBI reviewer link:* [*https://dataview.ncbi.nlm.nih.gov/object/PRJNA1337292?reviewer=gi2e8eg27s1878r0mnra0rcgkl*](https://dataview.ncbi.nlm.nih.gov/object/PRJNA1337292?reviewer=gi2e8eg27s1878r0mnra0rcgkl)*. Individual isolate accession numbers are listed in supporting information (S1 Table). The Data Availability Statement has been updated accordingly.*

Figure Resubmissions:

While revising your submission, we strongly recommend that you use PLOS’s NAAS tool (https://ngplosjournals.pagemajik.ai/artanalysis) to test your figure files. NAAS can convert your figure files to the TIFF file type and meet basic requirements (such as print size, resolution), or provide you with a report on issues that do not meet our requirements and that NAAS cannot fix.

After uploading your figures to PLOS’s NAAS tool - https://ngplosjournals.pagemajik.ai/artanalysis, NAAS will process the files provided and display the results in the "Uploaded Files" section of the page as the processing is complete. If the uploaded figures meet our requirements (or NAAS is able to fix the files to meet our requirements), the figure will be marked as "fixed" above. If NAAS is unable to fix the files, a red "failed" label will appear above. When NAAS has confirmed that the figure files meet our requirements, please download the file via the download option, and include these NAAS processed figure files when submitting your revised manuscript.

**Response:** *This has been done for all the figures.*
